# How to Apply the Soda Remedy in Burns and Scalds

**Published:** 1882-09

**Authors:** 


### HOW TO APPLY THE SODA REMEDY IN BURNS AND SCALDS.

It is now many years ago (see the London *Medical Gazette* of of March, 1844,) that the author of this paper, while engaged in some investigations as to the qualities and effects of the alkalies in inflammations of the skin, etc., etc., was fortunate enough to discover that a saline lotion, or *saturated* solution of the bicarbonated soda in either plain water or camphorated water, if applied speedily, or as soon as possible, to a burned or scalded part, was most effectual in immediately relieving the acute burning pain; and when the burn was only superficial, or not severe, removing all pain in the course of a very short time; having also the very great advantage of cleanliness, and, if applied at once, of preventing the usual consequences—a painful blistering of the skin, separation of the epidermis, and perhaps more or less of suppuration.

For this purpose, all that is necessary is to cut a piece of lint, or old soft rag, or even thick blotting paper, of a size sufficient to cover the burned or scalded parts, and to keep it constantly well wetted with the soda lotion so as to prevent its dying. By this means, it usually happens that all pain ceases in from a quarter to half an hour, or even in much less time.

When the main part of a limb, such as the hand and fore-arm or the foot and leg, has been burned, it is best, when practicable, to plunge the part at once into a jug, or pail, or other convenient vessel filled with the soda lotion, and keep it there until the pain subsides; or the limb may be swathed or encircled with a surgeon's cotton bandage previously soaked in the *saturated* solution, and kept constantly wetted with it, the relief being usually immediate, provided the solution be saturated and cold.

What is now usually sold as bicarbonate of soda is what I have commonly used and recommended ; although this is well known to vary much in quality according to where it is manufactured—but it will be found to answer the purpose, although probably Howard's is most to be depended on, the common carbonate being too caustic. It is believed that a large proportion of medical practitioners are still unaware of the remarkable qualities of this easily applied remedy, which recommends itself for obvious reasons.—F. Peppercorne, *in Popular Science Monthly for March.*

